# The chemistry and efficacy benefits of polysaccharides from *Atractylodes macrocephala* Koidz

**DOI:** 10.3389/fphar.2022.952061

**Published:** 2022-08-25

**Authors:** Congying Liu, Shengguang Wang, Zedong Xiang, Tong Xu, Mengyuan He, Qing Xue, Huaying Song, Peng Gao, Zhufeng Cong

**Affiliations:** ^1^ College of Pharmacy, Shandong University of Traditional Chinese Medicine, Jinan, China; ^2^ Shandong First Medical University Affiliated Shandong Tumor Hospital and Institute, Shandong Cancer Hospital and Institute, Jinan, China

**Keywords:** *Atractylodes macrocephala* Koidz., polysaccharide, chemical composition, biological activity, application

## Abstract

*Atractylodes macrocephala* Koidz (AM), traditional Chinese medicine (TCM) with many medicinal values, has a long usage history in China and other oriental countries. The phytochemical investigation revealed the presence of volatile oils, polysaccharides, lactones, flavonoids, and others. The polysaccharides from AM are important medicinal components, mainly composed of glucose (Glc), galactose (Gal), rhamnose (Rha), arabinose (Ara), mannose (Man), galacturonic acid (GalA) and xylose (Xyl). It also showed valuable bioactivities, such as immunomodulatory, antitumour, gastroprotective and intestinal health-promoting, hepatoprotective, hypoglycaemic as well as other activities. At the same time, based on its special structure and pharmacological activity, it can also be used as immune adjuvant, natural plant supplement and vaccine adjuvant. The aim of this review is to summarize and critically analyze up-to-data on the chemical compositions, biological activities and applications of polysaccharide from AM based on scientific literatures in recent years.

## Introduction

AM is the dry rhizome of *Atractylodes macrocephala* Koidz, which was first recorded in the ‘Shennong’s classic of materia medica’ and listed as the top grade. It is one of the common Chinese herbal medicines with high formula frequency in China, and it is used for treating diseases such as spleen hypofunction, loss of appetite, abdominal distension, diarrhoea, dizziness, and heart palpitations (Liu W. et al., 2021). There are more than 4755 Chinese patent medicines containing AM in China, which have been widely used because of their medicinal value and good clinical efficacy. AM (called “Baizhu” in China) is recorded in Chinese Pharmacopoeia (2020 version). AM has been prescribed as a traditional medicine in China, Korea and Japan for thousands of years (Editorial Committee of Flora of China, 2015; Kim et al., 2016; Liu J. et al., 2021). It is traditional Chinese medicine and edible plant having a health-protective effect. In recent years, a large number of related health care products have been listed, such as Jianwei Xiaoshi Tea, Huangqi Baizhu oral liquid, etc. At the same time, it is recorded in ancient Chinese medicine books that AM has the functions of removing blackness, replenishing qi and nourishing blood, whitening and nourishing, so it is fully used in beauty and medicinal food products. In the last few decades, various chemical components have been isolated from AM, and its chemical constituents include volatile oils, polysaccharides, lactones, flavonoids and other substances (Gu et al., 2019; Wang P. et al., 2020; Yang et al., 2021).

Polysaccharides are widely distributed in vegetation, animals, fungi and bacteria, and they have become hot spots in contemporary medical research. Many studies have shown that polysaccharides from AM are important biological active components. It possesses many pharmacological activities, such as enhancing immunity, antitumour, gastroprotective, hepatoprotective, intestinal health-promoting, hypoglycaemic, antioxidant, etc. (Zhu et al., 2018). This review is the first of its kind that gives a comprehensive discussion on the recent findings related to chemical composition, biological activities, pharmacological effects, and applications of polysaccharides from AM. It will construct a new foundation for further study of its mechanism and development of better therapeutic agent and healthy products.

## Chemical composition and structure

**FIGURE 1:**
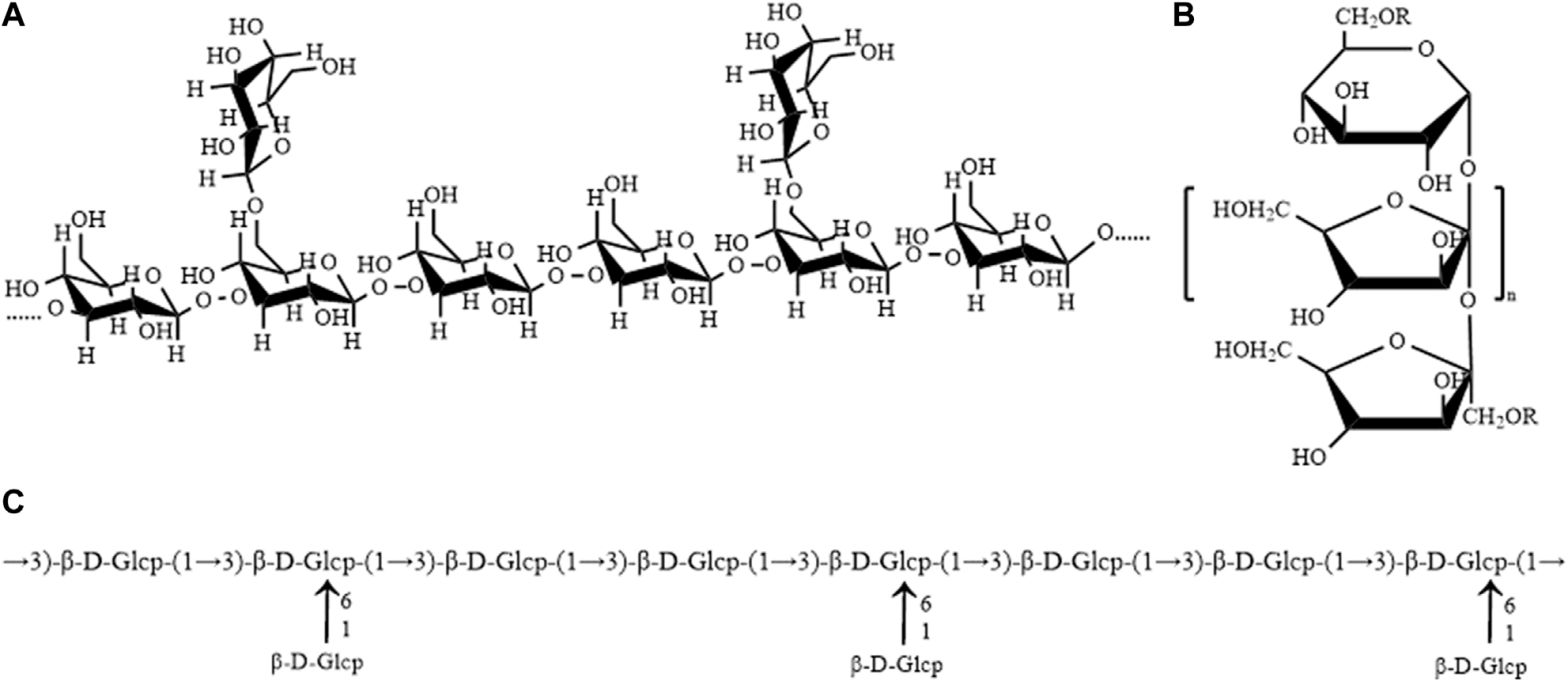
**(A)** Structure of UAM **(B)** Structure of inulin-type polysaccharide of AM **(C)** Structure of WAM (Wu et al., 2011; Wang, 2012; Lin et al., 2015).

Polysaccharide is one of the important active components of AM. Most studies have shown that polysaccharide from AM is a heteropolysaccharide composed of glucose, galactose, rhamnose, arabinose, mannose, galacturonic acid, xylose, etc. Among them, the proportion of Glc, Gal and Man is the highest, which is the main monosaccharide of polysaccharides from AM. The molecular weight is between 2.1 and 220 kDa. It is found that 1→3, 1→6, and 1→3.6 bond-type Glc is the main bond type of polysaccharides from AM, followed by 1→3 and 1→6 bond-type Gal. The chemical composition of polysaccharide from AM is shown in Table 1. The molecular weight, composition, molar ratio and sugar chain structure of polysaccharide from AM is different from those obtained from different extraction and purification methods. At the same time, polysaccharides with uncomplicated structures often do not have complex helical structures, and usually exist in the form of random coils in solution, and their conformations can be changed by metal ions, denaturants, and temperature. UAM is a homogeneous polysaccharide composed of glucose, mainly connected by β-D-1→3 and β-D-1→3.6, with simple structure and small molecular weight. Congo red test, viscometry, spectrophotometry, UV absorption and circular dichroism were used to analyze the behavior of UAM solution and the conformation of molecular chain in the solution. It was found that in the DMSO solvent system, UAM was in an irregular chain shape, without fixed form, and was easily affected by the external environment (Wang, 2012). Therefore, it is of great significance to control the extraction and purification methods of polysaccharides to ensure the structure and biological activity of AM. UAM, WAM and inulin-type polysaccharide of AM are three polysaccharides from AM with well-defined structures (Figure 1).

**TABLE 1:** The chemical composition of polysaccharides from AM.

Name	kDa	Monosaccharide composition	Structural feature	Method	Ref
RAMP-1	32.8	Ara: Gal: Glc: Man = 1: 0.74: 1.88: 1.07	→3-β-Glc*p*-(1→,→3,6-β-Glc*p*-(1→,→6-β-Glc*p*-(1→,T-β-Glc*p*-(1→,→4-α-Gal*p*A-(1→,→4-α-Gal*p*A-6-OMe-(1→,→5-α-Araƒ-(1→,→4,6-β-Man*p*-(1→,→4-β-Gal*p*-(1→	ELSD, IR, PC, GC, GC-MS, FGC/SAW	Tan, (2010)
RAMP		Ara: Gal: Glc: Man = 1: 0.74: 1.88: 1.07		GC, GC-MS, FGC/SAW, IR	Tan, (2010)
RAMP-2	4.354	Man: GalA: Glc: Gal: Ara = 1.00: 8.58: 27.28: 3.68: 4.99		NMR, FT-IR, HPSEC-MALLS, TEM, HPLC, SEM, HPGPC	(Tan, 2010; Xue et al., 2020)
RAMP-3					Tan, (2010)
PAMPS		ribose (Rib): Ara: Rha: Man: Glc: Gal = 1: 4.3: 0.1: 5.7: 2.8: 2.2	1,6-Glc	GC-MS	Zeng et al. (2018)
PAMPS	220, 2.15	Glc: Man: Ara: Gal: Xyl: Rib: Rha = 10.0: 3.2: 0.85: 0.40: 0.35: 0.17: 0.10	1.3-→β-D-Gal*p*, 1.6→β-D-Gal*p*	GPC, GC-MS, NMR	Zhao et al. (2016)
UAM	2921	Glc	1→3-β-D, 1→3,6-β-D	HPLC, GC, NMR, IR, UV	Wang, (2012)
RAMPtp	2.325	Glc: Man: Rha: Ara: Gal = 10.00: 2.47: 1.75: 1.46: 0.81	1,3-D-Gal*p*, 1,6-D-Gal*p*	NMR, HPLC, GPC, SEM	Xu et al. (2017)
PAMK	4.1	Ara: Glc: Gal = 1.5: 5: 1	α-D-Gal*p*, α-D-Glc*p*, α-L-Araƒ	GC-MS, FT-IR, HPGPC, GC, NMR	Feng, et al. (2019c)
PAMK	4.7	Ara: Glc: Gal = 1: 1.25: 6.78	→3-β-D-Glc*p*-(1→3,6-β-D-Glc*p*-(1→6-β-D-Glc*p*-(1→,T-β-D-Glc*p*,→4-α-Gal*p*A-(1→,→4-α-Gal*p*A-OMe-(1→,→5-α-Araƒ-(1→,→4,6-β-Man*p*-(1→,→4-β-Gal*p*-(1→	GPC-RI-MALS, IC, MS	Guo et al. (2021)
APA	2.1	Ara: Glc = 1: 4.57		GC, FT-IR, HPGPC	Feng, et al. (2019a)
PAM	28.773	Rha: Glc: Man: Xyl: Gal = 0.3: 2.5: 1.5: 4.1: 1.5		GC-MS, FT-IR, HPGPC, GC, NMR	Wang, et al. (2014)
AMP	23.91	Man: GalA: Glc: Ara = 12.05: 6.02: 72.29: 9.64		HPLC, GPC	Feng et al. (2020)
AMP	8.374	Glc: Gal: Rha: Man = 7.36: 1.00: 3.05: 1.52	α-(1→4) glycoside bond, β-(1→4) glycoside bond	HPLC, GC	Ji et al. (2015a)
PRAM2	19.6	Rha: Xyl: Ara: Glc: Man: Gal = 1: 1.3: 1.5: 1.8: 2.1: 3.2		GC-MS, HPLC, HPLC, HPSEC	(Yan et al., 2015; Han et al., 2016)
AMP-B		Glc: Gal: Man: Ara: Rha = 3.0: 2.5: 1.3: 3.5: 1.0		GC-MS	Shan and Tian, (2003)
YPF-P	3.5	Man: Glc: Gal: Ara = 1: 239: 18.6: 19.3		HPLC-GPC, TLC	Wang et al. (2019b)
YY13008	6.545	fructose (Fru): Glc = 40: 1	(2→1)-β-D-Fuc*p*, the terminal is α-glucopyranosyl group	HPLC, GC, NMR, GPC-MALLS	Wang et al. (2015a)
RAMPS	109.4	Glc: Man: Ara: Gal: Xyl: Rib: Rha = 10.00: 3.20: 0.85: 0.34: 0.35: 0.17: 0.10	1→6-Glc	GPC, GC-MS, NMR, FT-IR	Fan et al. (2016)
PSAM-1	136	Rha: Ara: Man: Gal = 0.68: 3.38: 1: 4.20	α-configurations; β-configurations	HPLC-ELSD, NMR	Chi et al. (2001)
PSAM-2	104	Xyl: Ara: Gal = 9.33: 0.64: 1	β-configurations	HPLC-ELSD, NMR	Chi et al. (2001)
AM-1	31	Man	β-configurations	IR, UV, GC	Gu et al. (1992)
AM-2	11	Fru	β-configurations	IR, UV, GC	Gu et al. (1992)
AMAP-1	137.5	GalA, Ara, Gal, Rha	41.5% 1,4-Gal*p*A; 13.9% Gal*p*	HPGPC, HPLC-ELSD, FT-IR, GC-MS, NMR	Cui et al. (2020)
AMAP-2	161.9	GalA, Ara, Gal, Rha	52.7% 1,4-Gal*p*A	HPGPC, HPLC-ELSD, FT-IR, GC-MS, NMR	Cui et al. (2020)
AMAP-3	85.3	GalA, Ara, Gal, Rha	73.1% 1,4-Gal*p*A	HPGPC, HPLC-ELSD, FT-IR, GC-MS, NMR	Cui et al. (2020)
BZJP	5.465	Glc: Man: Xyl = 6.8: 3.0: 1.0	βXyl*p*-[(1→3)-βXyl*p*]_6_-(1→6)-α-Glc*p*-[(1→4)-α-Glc*p*]_40_-[(1→2)-α-Man*p*]_12_	GC-MS, NMR, IR	Yang, (2021)
BZ-3–1	56.5	Glc: Man: Rha: Arab:Xyl: Gal = 1.6: 0.3: 1: 2.79: 0.1:0.3		HPGPC, GC-MS	Ji et al. (2017)
BZ-3–2	55.9	Glc: Man: Rha: Arab: Gal = 8.24: 2.78: 0.34: 1: 0.8		HPGPC, GC-MS	Ji et al. (2017)
BZ-3–3	55.1	Glc: Man = 5.49: 1		HPGPC, GC-MS	Ji et al. (2017)
WAM-1		Glc: Gal = 3.01: 1		HPLC, GC, AFM	(Wu et al., 2012)
WAM	3.263	Glc: Gal = 3.01: 1	β-D-1→3-D-Glc*p*; β-D-1→3,6-Glc*p*	HPLC, GC-MS, NMR, IR, AFM	Wu et al. (2011)
Inulin-type polysaccharide of AM	2.265	Glc: Fru = 1:2	α-D-Glc*p*-[(1→2)-β-D-Fucƒ]n-1-(1→2)-β-D-Fucƒ(*n* = 3～20)	ESI-MS, NMR, IR, HPLC-ELSD	Lin et al. (2015)

## Biological activities

### Immunomodulatory activity

Polysaccharides from AM have an immunomodulatory effect, and it is also known as Biological Response Modifier. The immunomodulatory effect is one of the main research areas of polysaccharides from AM, and its immunomodulatory activity has been demonstrated by a large number of *in vitro* and *in vivo* research experiments. (Li R. et al., 2018; Zhu et al., 2018; Yin et al., 2019). The following will introduce the immunomodulatory effects of polysaccharides from AM from four aspects: immune organs, immune cells, immune molecules, other signaling pathways and intracellular messengers (Table 2). The immune organs are composed of central immune organs (including bone marrow, thymus and bursa in poultry) and peripheral immune organs (including spleen, lymph and mucosal immune system etc.). The immune organs are responsible for immunogenesis, differentiation, maturation, settlement, proliferation, and immune response. The thymus is the site of T lymphocytes differentiation and maturation. The spleen is the body’s largest immune organ and produces many lymphocytes, especially B lymphocytes (Jiang et al., 2010; Wang et al., 2012; Xu and Tian, 2015a). Therefore, the developmental status of immune organs can determine the body’s immune regulation level. Thymus index, spleen index and weight are usually used as the initial indicators of immune function. A large number of experiments have proved that polysaccharides from AM can significantly increase the spleen and thymus index and body weight of experimental animals.

**TABLE 2:** Immunomodulatory activity mechanisms of polysaccharides from AM.

Polysaccharide	Target	Experimental model	Action or mechanism	Ref
PAMK	T-cells	CTX-treated female mice	The spleen index, IL-2, IL-6, TNF-α, IFN-γ, CD28^+^, PLCγ-1, IP3R, NFAT, AP-1 mRNA (+); CD28^+^/IP3R/PLC γ-1/AP-1/NFAT signal pathway	Xiang et al. (2020)
PAMK	T-cells and B-cells	CTX-treated geese	The spleen index, T cells and B cells count (+); TLR4-MyD88-NF-κB signal pathway	Li et al. (2021a)
PAMK	T-cells	CTX-treated geese	The index of thymus and spleen, GM-CSF, IL-1β, IL-5, IL-6, IL-4 and IL-10 (+); relative mRNA expression of novel-mir2, CD25^+^ and CD28^+^(-); novel-mir2/CTLA4/CD28^+^/AP-1 signal pathway	Li et al. (2021b)
PAMK-NLC	Lymphocytes	BALB/c mice	Splenic lymphocyte number, IL-1β, IL-12, TNF-α, IFN-γ, CD3^+^CD4^+^, CD3^+^CD8^+^(+)	Liu et al. (2018b)
AMP	RAW264.7 cells		NO, TNF-α, IFN-c, NF-κB (+)	Ji et al. (2015b)
RAMP2	Treg cells	Female C57BL/6 mice	FoxP3, IL-10 and IL-2. mRNA expression, IL-10, IL-2, STAT5 phosphorylation level (+); IL-2/STAT5 signal pathway	Xue et al. (2020)
RAMPtp	SMLN lymphocytes/RAW264.7 cells	Oxen	PI3K-AKT, MAPKs, NO, IL-6, IL-10, IFN-α, CCL2, CCL5(+) NF-κB, Jak-STAT and calcium signalling pathways	(Fan et al., 2016; Xu et al., 2020a; Xu et al., 2020b)
AMAP-1	RAW264.7 cells		Promoting NO release	Cui et al. (2020)
AMAP-2	RAW264.7 cells		Promoting NO release	Cui et al. (2020)
RAMPStp	Lymphocytes		T cell number, CD4^+^, CD8^+^(+)	Sun et al. (2015)
polysaccharide extract of AM		Adult male and female Sprague Dawley rats	The indexes of spleen and thymus (+); the expression level of Caspase-3, Smac/DIABLO and HtrA2/Omi (-)	Guo et al. (2012)
PAM	DC		DCs maturation, TLR4 expression, IL-12, TNF-α(+)	Ji et al. (2015b)

Immune cells can be directly or indirectly involved in the body’s immune regulation, thus playing a role in the immune system. Immune cells include macrophages (Mφ), natural killer (NK) cells, mast cells, T cells, B cells, dendritic cells (DC) etc. (Yang J. T. et al., 2018). Mφ can phagocytose and eliminate foreign pathogens. Polysaccharides from AM can bind with macrophage-specific membrane receptors to activate the immune response, which has the effect of directly killing pathogens. In addition, Mφ can also secrete various cytokines to enhance the body’s immune response (Yang et al., 2010; Hou et al., 2017). T cells are the primary effector cells of cellular immunity, including helper T (Th) cells and cytotoxic T (TC) cells (Zhuang et al., 2020). Th cells help B cells differentiate and make antibodies or help Mφ destroy intracellular pathogens. TC cells exert immune regulation mainly through CD8^+^ T cells, inhibiting T cell proliferation, antibody synthesis and secretion. The stability of the CD4^+^/CD8^+^ ratio is an important marker for maintaining an effective immune response (Liu et al., 2021). B cells mainly produce antibodies and enhance humoral immunity. Polysaccharides from AM can directly or indirectly increase the number of T cells or B cells and play an immunomodulatory role. DC mainly migrates and delivers captured antigens to Th cells, promotes T cell polarisation, and participates in regulating B cell function (Kara et al., 2014). The main effect of polysaccharides from AM on DC is to promote the expression of its surface molecules and the secretion of cellular molecules, thus enhancing the immune function of the organism.

The effects of polysaccharides from AM on immune molecules are mainly through affecting cytokines and antibody molecules. Cytokines are multi-functional active protein molecules, often involved in immune regulation, inflammation, hematopoiesis and other biological processes (Butcher and Zhu, 2021). The main families of cytokines include interleukins (ILs), chemokines, colony-stimulating factors (CSF), granulocyte-macrophage colony-stimulating factors (GM-CSF), interferons (IFNs) and tumour necrosis factors (TNF). Among them, IL-2, GM-CSF, TNF, and IFN-γ are secreted by Th1 cells and related to tumour cells’ immune response. IL-4, IL-5, IL-6, and IL-10 are secreted by Th2 cells and related to tumour cells’ immune escape. The secretion of ILs is regulated by forkhead lineage-transcription factor (FoxP3), which can increase the expression of FoxP3 by promoting the phosphorylation of signal transducers and activators of transcription 5 (STAT5) (Chen, 2021; Schmetterer and Pickl, 2017). Therefore, cytokines can act directly or indirectly on immune cells and exert biological effects. The antibody is a kind of immunoglobulin that can specifically bind to the antigen. Its structure can be divided into IgG, IgA, IgM, IgE, and IgD, with a certain immune regulation effect (Wei et al., 2017).

Cyclophosphamide (CTX) is an alkylating agent widely used in immunotherapy and cancer chemotherapy. However, it has many side effects, such as immunosuppression, myelosuppression, leukopenia, oxidative stress, disruption of Th1/Th2 balance and induction of decreased absolute T and B cell counts (Bordbar et al., 2011; Deng et al., 2018; Sun et al., 2018; Yesillik and Gupta, 2020; Boucher et al., 2021). Xiang et al. (Xiang et al., 2020) studied the mechanism of polysaccharides from AM in alleviating the decline of T cell activation induced by CTX. The results showed that polysaccharides from AM could significantly improve spleen indexes, balance Th1/Th2 ratio, alleviate abnormal shape and death of spleen cells, and increase the expression levels of IL-2, IL-6, TNF-α, and IFN-γ mRNA in serum of mice. Li et al. (Li et al., 2021a) also proved that polysaccharides from AM could alleviate the immunosuppressive effect of CTX on the goose. Sun et al. (Sun et al., 2015) proved that RAMPStp could promote the proliferation of T cells, promote the lymphocytes to enter S and G2/M phases, and significantly increase the percentage of CD4^+^T cells and CD8^+^T cells.

In addition, polysaccharides from AM can also affect the activities of intracellular messenger substances and related signal pathways. Nitric oxide (NO) can directly act on Mφ as a cytotoxic effector molecule to inhibit cancer cell immune response and improve the body’s immunity (Guzik et al., 2003). Ca^2+^ acts as the second messenger of the inositol phospholipid metabolic pathway. It is involved in the synthesis of lymphocytes DNA and cells growth, differentiation, and proliferation, thus regulating the body’s immune function.

Cyclic AMP (cAMP) and cyclic GMP (cGMP) are involved in cells activation and lymphocytes differentiation (Hou et al., 2017). Mitogen-activated protein kinase (MAPK) is the main signal pathway of cells oxidative stress and injury, including extracellular signal-regulated kinase (ERK), c-Jun N-terminal kinase (JNK) and p38, which can regulate cell growth, differentiation, and cell pathology (Kumar et al., 2015). CD28^+^ is an essential second signal in T cell activation by inducing phospholipase Cγ-1 (PLCγ-1) phosphorylation, which causes PLCγ-1 to hydrolyze inositol phospholipids on the membrane to generate inositol 1,4,5-trisphosphate receptor (IP3R) and diacylglycerol (DAG) (Boucher et al., 2021). DAG activates the protein kinase C (PKC) signalling pathway, which activates the phosphorylation of T nuclear factor (NFAT), activating protein 1 (AP-1) and nuclear factor κappa B (NF-κB). After being regulated by apoptosis-related gene B cell lymphoma-2 (Bcl-2), the Caspase family can directly destroy cell structure and induce mitochondrial apoptosis (Häcker, 2018). Related studies have found that Smac/DIABLO and Omi/HtrA2 are present in mitochondria, which can be released from mitochondria into cytoplasm when the cell structure is disrupted, thus serving as a marker of mitochondrial apoptosis (Grosser et al., 2021; Vega-Naredo et al., 2021).

Toll-like receptor 4 (TLR4) signalling is a classical pathway of polysaccharide mediated immunoregulation (Li et al., 2022; Zhao et al., 2022). Myelogenous differentiation factor 88 (MyD88) is the key downstream signal ligand of the TLR4 signalling pathway (Bates et al., 2020), which can induce resting nuclear transcription factor NF-κB to enter the cell nucleus, activate related gene transcription, promote the expression of inducible nitric oxide synthase, NO and cytokines, and activate the T cells to produce an immune response. Li et al. (Li B. X. et al., 2019) explored whether polysaccharides from AM could regulate mouse spleen function through the TLR4 signalling pathway. The results showed that the expressions of TLR4, MyD88, tumour necrosis factor receptor-associated factor 6 (TRAF6), TRAF3, and NF-κB mRNA in the spleen of mice in the polysaccharides of AM group were significantly increased. Therefore, the results suggest that polysaccharides from AM can act on the TLR4-MyD88-NF-κB signalling pathway and play an immune regulatory role.

**FIGURE 2:**
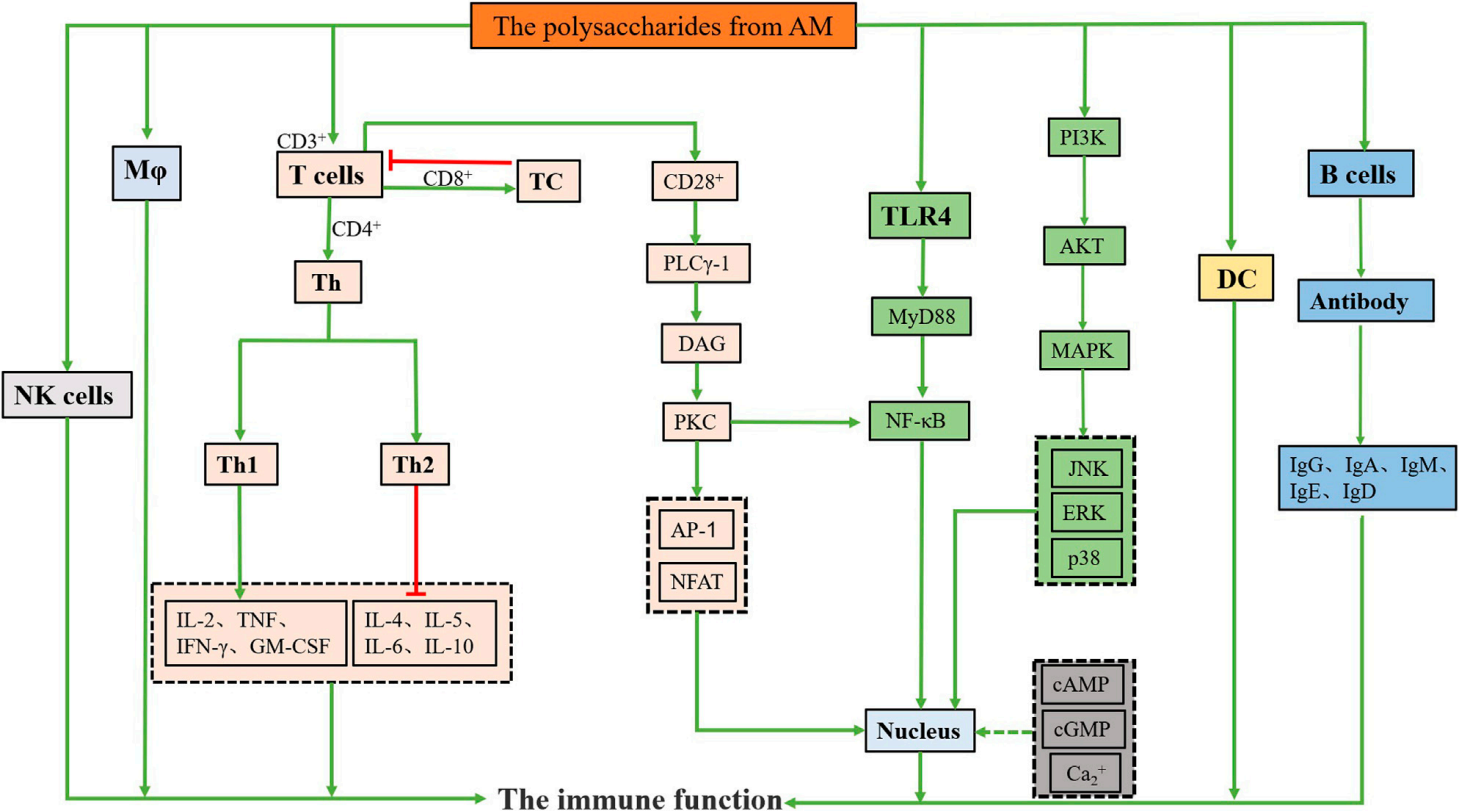
Immunomodulation activity mechanisms of polysaccharides from AM.

In conclusion, polysaccharides from AM can increase the weight of the spleen and thymus, reduce the damage to the immune organs, activate the immune cells, promote phagocytic activity, lymphocyte proliferation, associated cytokine secretion, antibody production, and improve the body’s immune system (Guo et al., 2012; Suabjakyong et al., 2015; Zhu et al., 2018). The related mechanisms are shown in Figure 2. These results suggest that polysaccharides from AM have potential as a therapeutic agent for as an immunomodulatory agent.

### Antitumour activity

Cancer is one of the major threats to human health. Radiotherapy and chemotherapy are currently widely used in cancer treatment, but most drugs used to treat cancer have serious side effects. Therefore, it is necessary to find new drugs or methods to replace the traditional cancer therapies (Yu et al., 2020). Plant polysaccharides are bioactive macromolecular substances extracted from plants, which have been proved to have antitumour activity with a high safety profile and alleviate the side effects of chemotherapy (Tian et al., 2017; Yu et al., 2018; Chen and Huang, 2019; Liu and Huang, 2019). It has been reported that *Astragalus* polysaccharide (Li et al., 2020a), *Hericium erinaceus* polysaccharide (Liu et al., 2020), *Angelica sinensis* polysaccharide (Yang J. et al., 2018) and *Lentinus edodes* polysaccharide (Xu et al., 2018) have antitumour activity. The polysaccharides from AM showed extensive antitumour activity on many kinds of tumour cells, such as oesophagal cancer, liver cancer, malignant glioma, colorectal cancer and lung cancer cells (Kou et al., 2017; Zhu et al., 2018). The anticancer activity is mediated via inducing tumour cell apoptosis, inhibiting tumour cell migration, invasion and metastasis, regulating the tumour microenvironment and immune function, and inhibiting tumour cell proliferation (Chen and Huang, 2018) (Table 3).

**TABLE 3:** Antitumour activity mechanisms of polysaccharides from AM.

Polysaccharide	Glycosidic bond	Target	Experiment	Action or mechanism	Ref
AMPs		Glioma C6 cells	*In vitro*	Mitochondrial membrane potential (-); Cyt-C, Caspase-9, Caspase-3, PARP (+)	Li et al. (2014)
APA	Ara: Glc = 1.00: 4.57; pyranose rings; α-type and β-type glycosidic linkages	Eca-109 cells	*In vitro*	Bcl-2, mitochondrial membrane potential (-); Bax, Cyt-C, Caspase-9, Caspase-3 (+)	Feng et al. (2019a)
AMP		Gastric cancer cells SGC-7901	*In vitro*	Bax, p53 mRNA (+); Bcl-2 (-)	Wang et al. (2015b)
PAM		Hepatocellular carcinoma cells	*In vitro*	AKT/GSK-3β phosphorylation (-), β-catenin and MMP2 protein expression (-); Wnt/β-catenin signalling pathway	Zhu et al. (2019)
PAM		Colorectal cancer cells	*In vivo*	IL-6, IFN-λ, TNF-α, NO (+); MyD88/TLR4 signalling pathway	Feng et al. (2019b)
AMP		Mouse colon cancer CT26 cells	*In vitro*	TNF-α, IL-2, CD4^+^/CD8^+^ (+); MDSCs(-); TLR4 signaling pathway	Feng and Yang, (2021)
AMP		Lung cancer cells	*In vivo*	IgG, IgA, IgM, CD3^+^, CD4^+^, CD4^+^/CD8^+^ (+)	Yin and Wang, (2019)
PAMK	α-D-Galp, α-D-Glc*p* and α-L-Araƒ	H22 hepatocarcinoma cells	*In vivo*	Arresting the H22 tumour cells at the S phase	Feng et al. (2019c)

The apoptosis of tumour cells induced by TCM includes two signalling pathways (Jearawuttanakul et al., 2020; Obeng, 2021): the exogenous pathway, activated by extracellular death signals. The other is the endogenous pathway, also known as the mitochondrial apoptosis pathway, in which mitochondria induce apoptosis in tumour cells. The mitochondrial apoptosis pathway mainly mediates the apoptosis of tumour cells induced by polysaccharides from AM.

**FIGURE 3:**
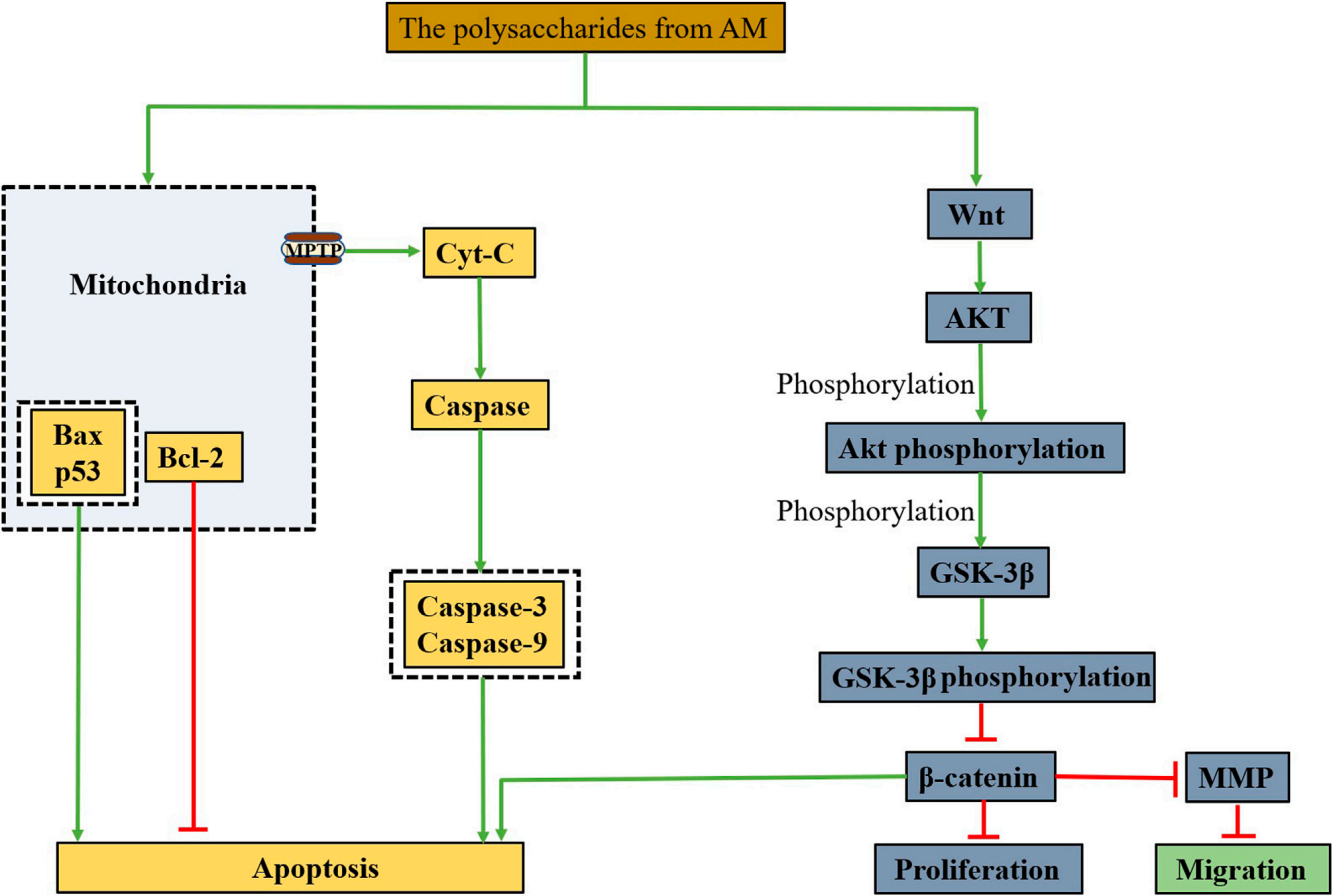
A possible mechanism by polysaccharides from AM to promote apoptosis, inhibit proliferation, migration through the mitochondrial apoptotic pathway and Wnt/β-catenin signaling pathway.

Caspase and polyadenosine diphosphate ribose polymerase (PARP) are the key enzymes of apoptosis, which can degrade the proteins in cancer cells and cause the cancer cells to die irreversibly (Okubo et al., 2017; Van Opdenbosch and Lamkanfi, 2019). Cytochrome C (Cyt-C) is present in mitochondria, and when the endogenous mitochondrial signalling pathway is activated, it causes mitochondrial outer membrane permeation (MOMP), and Cyt-C enters the cytoplasm to initiate Caspase cascade reaction and induce apoptosis (Orzalli and Kagan, 2017; Carneiro and El-Deiry, 2020). In addition, Bcl-2-associated X protein (Bax) has a pro-apoptotic effect, while Bcl-2 inhibits apoptosis. Feng et al. (Feng et al., 2019a) conducted structural analysis and *in vitro* cell experiments on the prepared alcohol-soluble polysaccharide and found that APA at a dose of 2 mg/ml could inhibit the proliferation of Eca109 cells by 74.63%, indicating that APA may reduce MOMP, increase Cyt-C, Caspase-9 and Caspase-3 by changing the expression of Bcl-2/Bax, and induced Eca109 cell apoptosis, as is shown in Figure 3.

In addition, polysaccharides from AM can inhibit the migration, invasion and metastasis of tumour cells by regulating related signal pathways. The secretory glycoprotein (Wnt)/β-catenin signalling pathway is a classic pathway of the Wnt pathway, including the Wnt protein family, glycogen synthesis kinase 3β (GSK-3β), adenomatous polyposis coli (APC), Axin, β-catenin and other key proteins (Pai et al., 2017; Zhang et al., 2017). Among them, β-catenin regulates transcriptional activity in cancer cells is related to the proliferation and invasion of cancer cells (Russell and Monga, 2018). AKT/GSK-3β is the upstream regulator of β-catenin in the Wnt pathway (Jain et al., 2017). When the Wnt signalling pathway is activated, AKT is phosphorylated to form phosphorylated AKT with kinase activity, which can inactivate GSK-3β by phosphorylating GSK-3β to form phosphorylated GSK-3β, causing reduced degradation of intracellular β-catenin to promote apoptosis of cancer cells (Katoh and Katoh, 2017; Zhang et al., 2017). Furthermore, it has been shown that matrix metalloproteinase (MMP) is a downstream target of the Wnt/β-catenin signalling pathway and participates in the migration of tumour cells (Katoh and Katoh, 2017; Zhang et al., 2017), in which MMP-2 is the dominant molecule in the metastasis of hepatoma cells, its overexpression is associated with metastasis of hepatoma cells (Dou et al., 2016). Zhu et al. (Zhu et al., 2019) explored the mechanism of PAM on proliferation and invasion of hepatoma cells *in vitro*. They found that PAM may inhibit proliferation and invasion of hepatoma cells by regulating the Wnt/β-catenin signal pathway (Figure 3).

Studies have shown that the occurrence of cancer is related to the characteristics of cancer cells themselves and influenced by the internal environment in which they live (Klein-Goldberg et al., 2014). The immune cells are important components of the tumour microenvironment, which can enhance the body’s immune function and inhibit the immune escape of tumour cells (Merlo et al., 2016). Myeloid-derived suppressor cell (MDSC) is one of the most important immunosuppressive cells. It has been found that there are a lot of MDSCs in tumour tissue, which can inhibit the activation of T cells in many ways and promote the adaptive growth of tumour cells (Hawila et al., 2017). Feng et al. (Feng et al., 2019b) suggested that PAM could promote lymphocyte proliferation and stimulate immune cells as an immune enhancer. The mechanism of action was investigated, and it was found that PAM could enhance T cells production of cytokines IL-6, IFN-λ, TNF-α, and NO, increase the CD4+/CD8+ ratio, and reduce the level of MDSCs through TLR4/MyD88 signalling pathway.

The cell growth process is divided into four stages: G1, S, G2, and M (Ye et al., 2020). Anticancer drugs can inhibit the growth of cancer cells by inhibiting their cell cycle. Feng et al. (Feng Z. et al., 2019) obtained the crude polysaccharide of AM by water extraction and alcohol precipitation method and purified by ultrafiltration. Finally, a neutral heteropolysaccharide PAMK with a molecular weight of 4.1 kDa was obtained by HPGPC, FT-IR, GC, and NMR. PAMK arrested the cell cycle in the S phase, prevented the chromosome replication, inhibited their division, and thus inhibited the proliferation of cancer cells.

In conclusion, polysaccharides from AM have good antitumor activity, which is mainly mediated via inducing tumour cell apoptosis, inhibiting tumour cell migration, invasion and metastasis, regulating the tumour microenvironment and immune function, and inhibiting tumour cell proliferation. However, the structure of polysaccharides from AM are complex, and it is difficult to explain its structure-activity relationship. The mechanism involved in the antitumour activity of polysaccharides from AM should be further studied. In addition, the antitumour activity of polysaccharides from AM was mainly focused on *in vitro* experiments. Thus, more studies should be conducted to evaluate its antitumour activity and safety profile in the relevant *in vivo* models.

### Gastroprotection and intestinal health promotion

AM has a long history of use as a herb with health-protective effects mainly for digestive system diseases such as food intake reduction, bloating and diarrhoea. It has the functions of invigorating the spleen, improving gastrointestinal function, repairing gastrointestinal mucosal damage and regulating intestinal microecology (Wang J. et al., 2019; Yang et al., 2020; Wen et al., 2021). Many papers report that plant polysaccharides (Wang W. et al., 2020; Sun et al., 2020; Yan et al., 2020; Cui et al., 2021) can effectively treat gastrointestinal diseases. The gastroprotective activity of polysaccharides from AM is mediated *via* the regulation of intestinal flora and related signalling pathways, the synthesis and release of gastrointestinal hormones and neurotransmitters, and oxidative stress response. The details are shown in Table 4.

**TABLE 4:** Gastroprotective and intestinal health promoting mechanisms of polysaccharides from AM.

Polysaccharide	Function	Model	Glycosidic bond	Action or mechanism	Ref
PAM	Regulating the flora of intestinal disorders	Male SD rats	xylose	Activating and accelerating the growth of the intestinal flora	Wang et al. (2014)
PAMPS	Accelerating migration of IEC-6 cells and treating GI mucosal injury	IEC-6 cells	Rib: Ara: Rha: Man: Glc: Gal = 1.0: 4.3: 0.1: 5.7: 2.8: 2.2	Intracellular polyamines and Kv1.1 channel expression and activity (+), [Ca^2^+]cyt (+)	Zeng et al. (2018)
PAMK	Repairing the damage to the gastrointestinal mucosa	*Anser cygnoides*		Promoting the development of small intestinal villi, polyamine content (+); endotoxin, CRP, IL-1β, IL-6, TNF-α(-)	Li et al. (2021c)
AMKP	Strengthening the small intestine epithelial barrier, protecting the gastrointestinal mucosa	IEC-6 cells	The Fructose unit β (2→1) is a fructan with a terminal end connected by an α (1→2) glucoside bond	Cell Ca^2+^ level (+), expression of E-cadherin, α-catenin, β-catenin (+)	Luo et al. (2021)
RAMPtp	Protecting the intestinal barrier dysfunction induced by DSS.	IPEC-J2	Linked by 1,3-linked β-D-Gal*p* and 1,6-linked β-D-Gal*p* residues	Phosphorylated STAT2 protein (p-STAT2) (-); the expression of TJ protein (+), the proliferation and survival of IECs (+)	Zong et al. (2021)
AMP	Protecting intestinal mucosal barrier function	SPF-grade male C57BL/6 mice	1,3-β-D-Galp and 1,6-β-D-Galp	TNF-α, IL-6, IL-1β mRNA expression (-), phosphorylated STAT2 protein (p-STAT2) (-); the expression of TJ protein (+)	Kai, (2021)
AMP	Treating UC, ameliorating colonic injury	Forty specific-pathogen-free (SPF)-grade male C57BL/6J mice		The expression of claudin-1, occludin, ZO-1 was regulated; the expression of mucin-2 (+)	Feng et al. (2020)
AMP	Improving small bowel barrier function	Weanling pigs		The expression of CDKN1A gene was regulated to inhibit the proliferation of jejunal epithelial cells; the expression of ZO-1, claudin-1 and occludin mRNA (+)	(Xie et al., 2019; Chen et al., 2020)
AMP	Repairing the mucosal injury caused by *escherichia coli* diarrhea	Clean ICR male mice		Regulation of gene expression of TGF-β1 and EGFR.	Shang, (2017)
AMP	Relieving spleen deficiency and improving gastrointestinal function	SPF-grade male mice		GAS, SS (+); VIP (-)	Li et al. (2018b)
AMP with content over 95%	Treatment of stress ulcer	Male SD rats		SOD activity (+), Bcl-2 expression (+); Bax expression (-), MDA content (-)	Cao & Bai, (2016)
YY11901	Relieving the gastrointestinal mucosa injury	IEC-6 cells			Wang, (2018)

Polysaccharides from AM can regulate the gene signalling pathway to increase tight junction (TJ) protein expression. Gastrointestinal mucosa is an epithelial barrier formed by intercellular connection and interaction to resist the invasion of foreign harmful substances. TJs, mainly composed of membrane proteins such as occludin, claudins and zona occludens 1 (ZO-1), are important components of the intestinal epithelial barrier and play an important role in maintaining intestinal permeability, tissue differentiation and homeostasis (Ren et al., 2019; Tan et al., 2019). Polyamines regulate cell junctions by influencing cell signalling. TJs are located at the top of the junction complex, and the adhesive junctions below are rich in cadherin. Cadherin is a Ca^2+^ dependent transmembrane protein. E-cadherin is located in the adherens junctions of the intestinal mucosa and binds to intracellular α, β, and γ connexins (catenin) for the formation and regulation of epithelial barriers (Shafraz et al., 2020). Kv pathway (Fellerhoff-Losch et al., 2016) is the decisive factor to increase Ca^2+^ level. Long non-coding RNA are non-protein coding transcripts (Noh et al., 2018), used for the regulation of transcriptional genes, and play an important role in describing intestinal barrier function (Batista and Chang, 2013; Quinn and Chang, 2016; Kok and Baker, 2019; Hanning et al., 2021). It protects intestinal barrier function by blocking phosphorylated signal transduction and nuclear translocation of transcriptional activator 2 (STAT2). CDKN1A gene inhibits jejunal epithelial cells apoptosis by regulating the activity of cyclin-dependent kinases (Kreis et al., 2019). AMKP can increase polyamines in cells, enhance the expression and activity of the Kv1.1 pathway, increase the level of Ca^2+^, stimulate accelerated cell migration, increase the expression of mucosal junction proteins, and accelerate the healing of intestinal injury (Luo et al., 2021). Short-chain fatty acids are intestinal microbial metabolites. Their abnormal changes are a significant feature of ulcerative colitis (UC) at the metabolic level (Feng et al., 2018; Paramsothy et al., 2019).

Polysaccharides from AM can also regulate the synthesis and release of gastrointestinal hormones and neurotransmitters and improve gastrointestinal function. The hormones or peptides produced by the gastrointestinal tract can be divided into three groups: gastrointestinal hormones, gastrointestinal neuropeptides and gastrointestinal growth factors (Hayati et al., 2021). Gastrin (GAS) and vasoactive intestinal peptide (VIP) are two brain-gut peptides. GAS can stimulate gastrointestinal movement; VIP mainly regulates gastrointestinal relaxation and gastrointestinal sphincter relaxation. Somatostatin (SS) is a peptide hormone that inhibits gastric acid secretion. The gastrointestinal growth factor can regulate the process of wound repair caused by intestinal inflammation. AMP can promote the expression of cytokines related to intestinal mucosal repair, such as pro-epidermal growth factor (EGF) and transforming growth factor-β1 (TGF-β1), to alleviate the injury of gastrointestinal mucosa (Shang, 2017).

Polysaccharides from AM can improve the antioxidant capacity of gastric mucosa, alleviate oxidative stress, regulate the expression of apoptosis-related genes, and reduce the damage of free radicals to the gastric mucosa. AMP alleviates exercise stress ulcers in mice by increasing Superoxide dismutase (SOD) activity, up-regulating Bcl-2 expression, down-regulating Bax expression and malondialdehyde (MDA) content (Cao and Bai, 2016).

PAM can further improve the intestinal microenvironment by regulating the growth of intestinal flora and play a protective role in the gastrointestinal tract. Studies have found that it can promote the proliferation of intestinal probiotics, inhibit the growth of harmful bacteria, and regulate the composition of intestinal flora (Wang et al., 2014).

In addition, YY11901, a polysaccharide isolated from AM by Wang (Wang, 2018), can alleviate the injury of the gastrointestinal mucosa, but the specific mechanism needs to be further studied. Thus, these results suggest that the representative compound AMP could be a promising structural template for the development of new gastrointestinal drug candidates.

### Hepatoprotective effect

**FIGURE 4:**
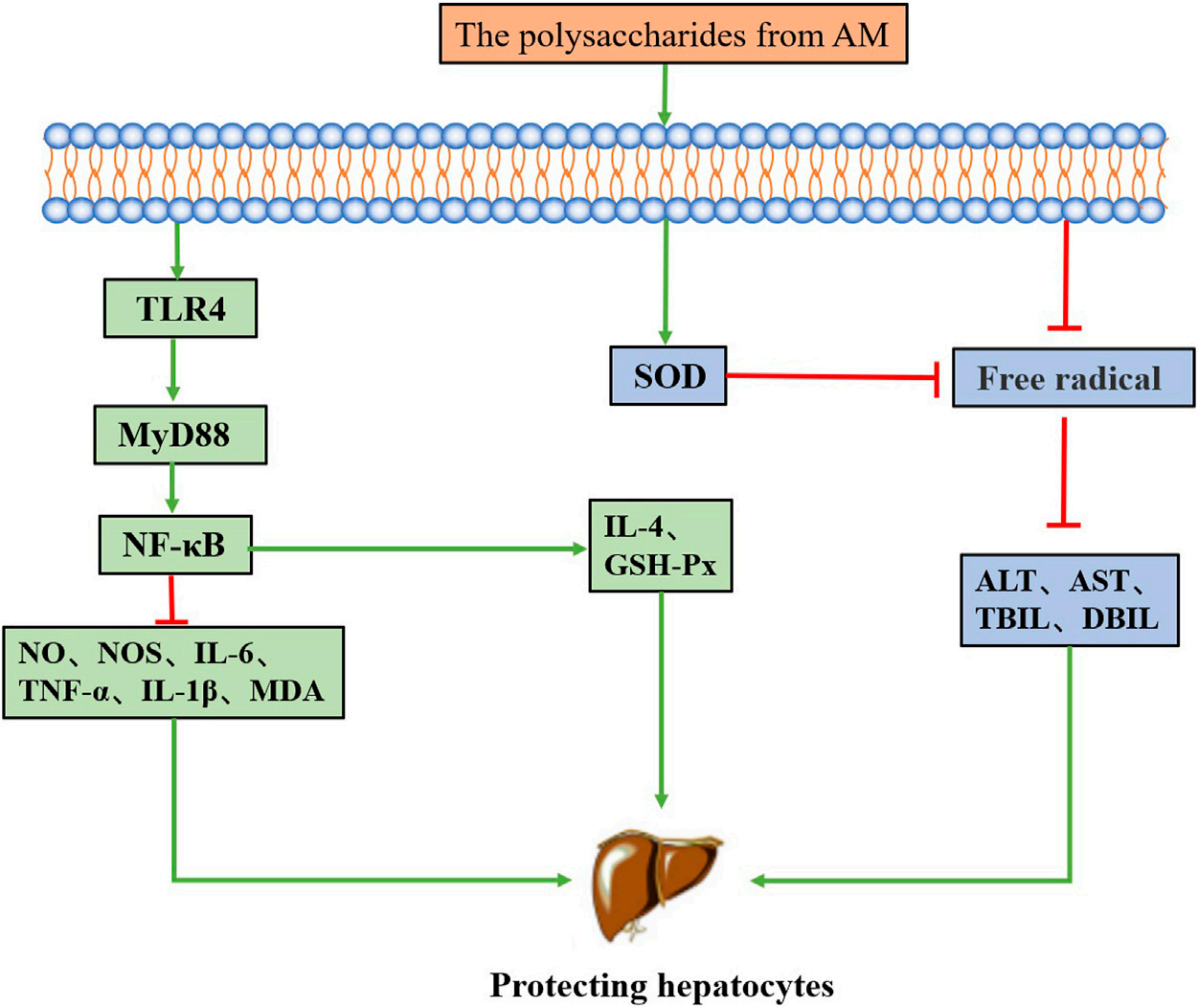
The mechanism of the hepatoprotective activity of polysaccharides from AM.

Liver injury (Qu et al., 2020) is an important pathological process in liver diseases, leading to fatty liver, liver ischemia-reperfusion injury (IRI), liver cirrhosis, fibrosis and even liver cancer. Studies have shown that plant polysaccharides (Zhang et al., 2021) have hepatoprotective activity and also have the advantages of wide distribution, low toxicity, and low price. It is considered to be an important natural resource for alleviating liver injury. AM has hepatoprotective activity, and its main chemical constituent polysaccharides from AM are effective in the treatment of IRI, non-alcoholic fatty liver disease (NAFLD) and liver injury. Its mechanism of action mainly includes regulating related anti-inflammatory signaling pathways and relieving oxidative stress. The related mechanism of polysaccharides from AM alleviating liver injury is shown in Figure 4.

Oxidative stress produces large amounts of free radicals, which damage the membranes of liver cells through lipid peroxidation (Li et al., 2021b). Enzymes and proteins such as serum alanine transaminase (ALT), aspartate transaminase (AST), total bilirubin (TBIL) and direct bilirubin (DBIL) escape from hepatocyte membranes after injury so that these components can be used as markers of liver injury. The levels of MDA and SOD reflect the extent of lipid peroxidation damage in hepatocytes (Li et al., 2020b). It has been found that the occurrence and development of fatty liver are often accompanied by an abnormal increase in the content of triglyceride (TG) and free fatty acids (FFA) in liver tissue (Song et al., 2021). Han et al. (Han et al., 2016) isolated a polysaccharide PRAM2 from AM and found it to protect the acute liver injury induced by carbon tetrachloride in mice *via* alleviating the oxidative stress response. PRAM2 reduced the ALT and AST activity, reduced the nitric oxide synthase, NO and MDA contents, increased the SOD and glutathione peroxidase (GSH-Px) activity.

IRI is often accompanied by oxidative stress and reactive oxygen intermediates. It has been reported that oxidative stress may activate NF-κB to regulate inflammatory factors (Kuboki et al., 2007; Tóbon-Velasco et al., 2014). Jin et al. (Jin et al., 2011) found that polysaccharides from AM inhibited the IRI induced lipid peroxidation, suppressed the elevation of ALT, AST, TBIL, and MDA, decreased the NF-κB expression, and promoted the SOD activity. In addition, Guo et al. (Guo et al., 2021) found that PAMK alleviates lipopolysaccharide-induced hepatitis in mice by regulating the TLR4-MyD88-NF-κB signalling pathway, decreasing the IL-1β, IL-6, and TNF-α levels, increasing the IL-4 level and inhibiting GSH-Px and MDA levels. Qian et al. (Qian et al., 2019) also found that PAMK alleviates CTX-induced liver injury in goslings, which may be related to the regulation of the TLR4 signalling pathway.

NAFLD is a liver disease closely related to insulin resistance, with high incidence in Europe and America. It may induce diseases such as liver cirrhosis and liver cancer in severe cases. Che et al. (Che et al., 2017) found that polysaccharides from AM could effectively prevent and treat NAFLD, alleviate steatosis and vacuolar degeneration of liver cells, and reduce the levels of TG, FFA, ALT, and AST. Studies have shown that polysaccharides from AM are also associated with fatty acid transporter and apolipoprotein B100 in NAFLD treatment (Xia et al., 2017).

Hence, polysaccharides from AM have certain hepatoprotective activity, and pharmacological studies have shown that it can effectively alleviate acute liver injury, IRI, hepatitis and NAFLD in mice. Further studies should be carried out to explore its activity in alleviating alcoholic fatty liver disease.

### Hypoglycaemic activity

**FIGURE 5:**
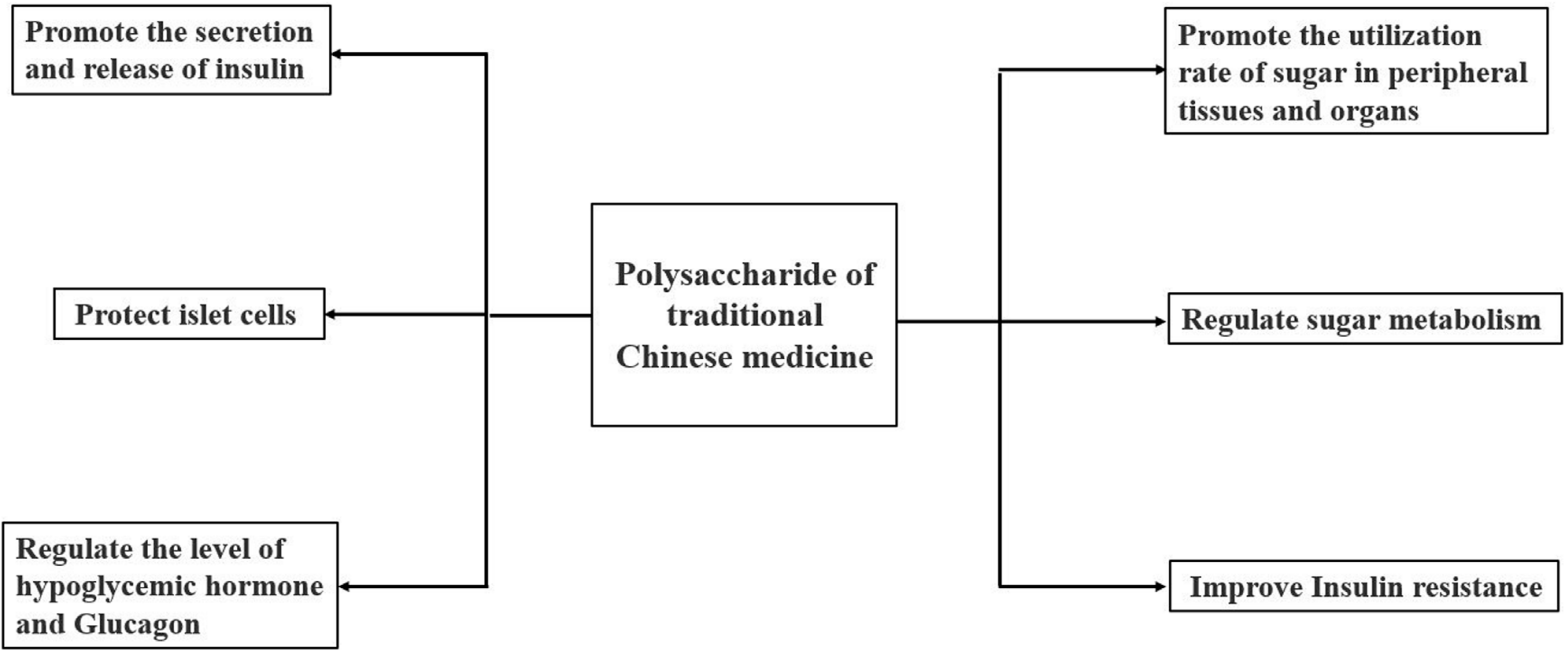
Hypoglycaemic mechanisms of polysaccharides from TCM.

Diabetes mellitus (Jiang et al., 2020) is a metabolic disease caused by long-term hyperglycemia due to insufficient insulin secretion and insulin dysfunction. TCM has significant hypoglycaemic activity with few side effects. The mechanism of hypoglycaemic activity of polysaccharides may be related to the protection of islet cells, the promotion of insulin secretion and release, the improvement of insulin resistance, the regulation of glucose metabolism, the regulation of hypoglycaemic hormone and glucagon levels, and the promotion of glucose utilisation in peripheral tissues and organs (Dong et al., 2019; Zhong et al., 2021). The mechanisms are shown in Figure 5.

A study found that alloxan selectively destroys islet β cells, making insulin less available and causing blood sugar to rise (Drikvandi et al., 2020). Shan et al. (Shan and Tian, 2003) screened the purified polysaccharide AMP-A and AMP-B from AM for their hypoglycaemic activity. AMP-A had no effect, whereas AMP-B had the effect in alloxan-induced diabetic rats. AMP-B can effectively alleviate the atrophy of the thymus and islet in diabetic rats, which may be through inhibition of islet β cell injury induced by alloxan. Li et al. (Li et al., 2015) found that polysaccharides from AM can reduce fasting and postprandial blood glucose in db/db mice. Its mechanism may be related to improving the sensitivity of peripheral target organs to insulin, reducing plasma insulin levels, increasing insulin sensitivity index and improving insulin resistance.

In summary, the hypoglycaemic activity mechanism of polysaccharides from AM is the same as other plant polysaccharides. Its activity is mainly mediated *via* protecting islet cells, promoting insulin secretion or release, improving insulin resistance and promoting sugar utilisation by peripheral tissues and organs. In the future, we should continue to study the hypoglycemic mechanism of polysaccharides from AM, and focus on exploring its different action pathways and targets from other polysaccharides, so as to develop it into a specific drug for the treatment of diabetes.

### Other activities

In addition to the above-mentioned pharmacological activities, polysaccharides from AM also have antibacterial, neuroprotective, antiaging (Ma et al., 2006), antioxidant and other activities. The antibacterial activity of polysaccharides from AM was mainly manifested in inhibiting the growth of *Aeromonas* (Fan and Liang, 2017). Polysaccharides from AM also have antioxidant activity. Pu et al. (Pu et al., 2015) used ultrasound-assisted enzymatic extraction to obtain RAMP with antioxidant activity. The antioxidant activity was evaluated using DPPH free radical scavenging method, and the scavenging rate was up to 62.18%. The polysaccharides obtained from AM purified by DEAE column were selenized by Hou et al. (Hou et al., 2019) to obtain sAMP6. It possesses the strongest *in vitro* and *in vivo* antioxidant activity mediated *via* increasing the GSH-Px and SOD activities and reducing the MDA in experimental chickens. In addition, polysaccharides from AM also have a neuroprotective effect (Hu et al., 2014). It can significantly inhibit hypoxia-induced neuronal growth inhibition, mitochondrial injury and apoptosis. The mechanism of polysaccharides from AM is related to the decrease of Bax and Caspase-3, the increase of Bcl-2/Bax ratio, and the inhibition of mitochondrial apoptosis. In addition, the adverse reactions and side effects of polysaccharides from AM have not been found so far. In summary, polysaccharides from AM have numerous pharmacological activities, which provide a strong reference for the clinical application of AM in Chinese medicine.

### Structure-activity relationship

At present, the research on the structure-activity relationship of plant polysaccharides is a hotspot of polysaccharides. Plant polysaccharides have complex molecular structures, and differences in monosaccharide composition, molecular weight, spatial structure and chemical modification have important effects on the biological activity of polysaccharides (Lin and Huang, 2022).

The monosaccharide composition of polysaccharides from AM is related to its pharmacological activity. It has been reported that xylose could activate the proliferation of bifidobacteria and improve the intestinal microbial environment (Broekaert et al., 2011) (Wang et al., 2020) (Wang et al., 2014), found that PAM regulates the intestinal flora associated with xylose in its structure, which activates and accelerates the growth of intestinal flora. In addition, galacturonic acid is an important monosaccharide constituting PAMK, and related studies have found that galacturonic acid can activate complement, which may be an important reason for PAMK-mediated immune response (Huang et al., 2022). Studies have shown that spherical polysaccharide molecules containing Man and Gcl are more conducive to binding with receptors to trigger downstream signaling factors (Lombard et al., 1994). RAMP2 and PAM are spherical polysaccharides containing Man and Glc, and their immunomodulatory and anti-tumor effects may be related to the specific binding of monosaccharides Man and Glc to polysaccharide receptors on Mφ. It can be seen that the composition of polysaccharides determines its structural properties, and the structural properties determine its biological activity.

A specific spatial structure is a necessary condition for the biological activity of polysaccharides. The study found that the basis of the biological activity of polysaccharides is the single-stranded helical conformation of β-(1→3)-D-glucan (Saitô et al., 1991). UAM is a homogeneous polysaccharide composed of glucose, mainly connected by β-D-1→3 and β-D-1→3.6, with simple structure and small molecular weight. Its activity of inhibiting the growth of K562 cells may be related to its structure. Meanwhile, the side chain portion of the RG-I region in AMAP-1 and AMAP-2 was found to stimulate immune activity and significantly increase NO release in RAW264.7 cells (Broekaert et al., 2011; Cui et al., 2020). However, the current research on the spatial structure of polysaccharides from AM is still very superficial, and the regular structural pattern has not been summarized, and a large number of experiments are needed for supplementary verification.

The molecular weight is closely related to the activity of polysaccharides from AM. A study found that the immune activity of AMAP-1 and AMAP-2 was significantly higher than that of AMAP-3, which may be related to the higher molecular weight of the former and the lower molecular weight of AMAP-3 (Cui et al., 2020). *Sijunzi Decoction* consists of *Panax ginseng*, *Atractylodes macrocephala*, *Poria cocos Wolf* and *Glycyrrhiza uralensis*. A total of seven polysaccharides were isolated through experiments. Among them, BZ-three to one extracted from AM has the highest immune activity, which can significantly enhance the phagocytosis of RAW264.7 cells, which may be related to its highest molecular weight (Ji et al., 2017). It can be seen that polysaccharides with higher molecular weight may have stronger immune activity.

Studies have shown that the biological activity of polysaccharides from AM after chemical modification has been significantly improved. The structural modification of polysaccharides from AM mainly focuses on selenization, modification of functional groups and chelation. Selenium is an important trace element with many pharmacological activities such as anti-cancer, enhancing immunity and anti-inflammatory (Huang et al., 2020). Selenium polysaccharide is a compound in which selenium is bonded to active polysaccharide, which can play the dual functions of selenium and polysaccharide (Li J. et al., 2019; Zhou et al., 2020). It has been shown that selenium-modified polysaccharides from PAMK can enhance immune activity significantly promote the proliferation of lymphocytes and the secretion of related cytokines (Xu et al., 2014; Li W. et al., 2018). At the same time, Xu and Tian (Xu and Tian, 2015b) also found that the combination of PAMK and selenium could better improve the immune response of chickens with acute heat stress. And found that selenium can specifically regulate TNF-a and IFN-γ pathways, and PAMK can affect more cytokines. In addition, the modification and types of functional groups in polysaccharides have a significant impact on their pharmacological effects. The introduction of sulfuric acid groups changed both the primary and higher structures of polysaccharides from AM. Sulfation modification had better anticoagulation and B lymphocyte proliferative activities and showed a dose-response relationship. Jiang (Jiang, 2011) investigated the effects of unsulfated PAMK and sulfated PAMK on the proliferation of chicken peripheral blood lymphocytes through experiments, and found that sulfation could significantly improve the lymphocyte proliferation of PAMK in a dose-effect relationship. Wang (Wang, 2012) compared the antioxidant ability of hydroxymethylated AMP and the positive control acarbose on glycosidase, and found that hydroxymethylated AMP has a good inhibitory effect on glycosidase, and can be developed into a hypoglycemic drug in the future. In addition, polysaccharide-metal complexes are also an important modification method to enhance the activity of PAMK. Wu used the prepared PAMK-zinc complex as a feed additive to investigate its effect on the immune regulation function of immunosuppressed rats, and found that low-dose PAMK-zinc complexe could restore part of the immune function of rats, which may be related to the better absorption and utilization in rats. In conclusion, structural modification is an important way to improve the biological activity of polysaccharides from AM. However, due to the limitations of spatial structure testing technology, the research on the relationship between polysaccharides from AM structure and biological activity mechanism and the improvement of its structural activity are relatively rough, and the clinical trials are not sufficient, which limits its development and application.

In addition, there are related reports on the type modification of polysaccharides from AM preparations. Mesoporous silica nanoparticles (MSNs) are a kind of porous non-metallic material-based carriers, which have the advantages of high surface area, stability, targeting, and biocompatibility, and are widely used in drug control delivery. Based on PAMK’s fast metabolism and poor targeting, MSNs were prepared for loading PAMK. It was found that PAMK-MSNS can enhance the phagocytosis of macrophages and has good safety. Nanostructured lipid carriers have high drug loading capacity for lipophilic and hydrophilic drugs, good biocompatibility and high bioavailability. Liu et al. found that PAKM-NLC could promote the maturation and differentiation of bone marrow dendritic cells, and promote the secretion of IL-1β, IL-12, TNF-α, and IFN-γ, which is expected to be a good adjuvant for improving immune efficacy. It can be seen that the formulation modification of PAMK can also significantly improve its pharmacological activity. However, there still have certain limitations, and their stability is easily affected by environmental factors (such as temperature, pH value, etc.). At the same time, there are only a few studies on polysaccharides from AM as a drug carrier. Thus further studies should be carried out to explore the potential of developing polysaccharides from AM as a drug carrier.

It can be seen that there is a significant relationship between the structure of polysaccharides from AM and its pharmacological activity. However, the structure of polysaccharide is complex and different extraction processes may affect its structure and yield, resulting in structural instability. Therefore, in the future research, more emphasis should be placed on the optimization of the extraction process of polysaccharides from AM, and the connection between its primary structure and even its advanced structure and pharmacological activity should be deeply analyzed by using advanced structure identification methods. At the same time, different techniques should be used to modify the structure of polysaccharides from AM to improve its structural properties and enhance its pharmacological activity.

## Applications

AM is a commonly used Chinese medicine in clinics and belongs to dietary supplements. It has good therapeutic effects on protecting gastrointestinal mucosa, balancing intestinal flora, relieving cardiovascular disease and regulating immune system function.

Polysaccharides from AM have the name of immune adjuvant, which is the basis of the medicinal effect of AM to exert immune regulation function. It has been proved that polysaccharides from AM have efficacy in regulating the body’s immunity and have a certain alleviating effect on the adverse reactions caused by the immune deficiency. Therefore, polysaccharides from AM can treat chemotherapy-induced adverse reactions such as weight loss, intestinal mucosa injury, and intestinal flora disturbance. It can protect the integrity of the intestinal barrier and maintain the stability of gastrointestinal function to alleviate the side effects caused by chemotherapy (Wang et al., 2018).

Due to their naturalness and versatility, natural plant supplements are safe and do not develop drug resistance. These are in line with the development trend of the modern food industry and are the hot topics of domestic and foreign animal husbandry research (Ali et al., 2021; Redhead et al., 2021). Polysaccharides from AM can significantly improve intestinal health and enhance the immune function of animals, promote the growth of animals, increase feed intake and reduce the feed-meat ratio. In addition, polysaccharides from AM also have good antibacterial, antiviral and antioxidant effects. These effects would improve the resistance capacity of animals to the diseases (Song et al., 2014). The development of polysaccharides from AM as an effective food supplement to animals is of great significance and is in line with the rational and sustainable development of antibiotic-free animal feed. However, it should be noted that the structure of polysaccharide has important implications for the generation of its pharmacological effects. The source and extraction method of AM may affect the structure of polysaccharides, since the composition of polysaccharides from different regions or extracted using different methods may not be the same and may affect the efficacy. At the same time, the dose of polysaccharides from AM used is not the same in all animals, and different developmental stages of animals may require different doses (Gao et al., 2019; Yang et al., 2019). Furthermore, only for extraction conditions, the laboratory studies are not sufficient to translate into its application in animal husbandry industry. Hence, The extraction procedures also should be optimised on an industrial scale.

Polysaccharides from AM can also be used as a vaccine adjuvant to enhance immunomodulatory effects. It has been found that the immunomodulation of polysaccharides from AM can be enhanced by structural modification or the preparation of nanoparticles. Liu et al. (Liu X. et al., 2018) combined PAMK-NLC with PAMK to prepare PAMK-NLC nanoparticles and found that PAMK-NLC could enhance the immune effect of PAMK, so it could be used as an adjuvant of OVA. In addition, oral or subcutaneous injection of AMP was found to enhance the immune response of mice to the foot-and-mouth disease (FMD) vaccine and can be used as an adjuvant of the FMD vaccine (Xie, 2012; Lu et al., 2016). Polysaccharides are chemically stable, readily available and easily modified, and are widely used in the development of targeted drug carriers. However, there are few studies on polysaccharides from AM as a drug carrier. Therefore, the potential of developing polysaccharides from AM as a drug carrier should be explored in depth.

## Conclusion and prospect

This review summarizes the research progress in chemical compositions, pharmacological activities and applications of polysaccharides from AM. It is the main chemical component present in AM and possesses significant pharmacological activities. Polysaccharide from AM is a heteropolysaccharide, mainly composed of monosaccharides such as Gal, Rha, Ara, Man, GalA, and Xyl. In addition, it has immunomodulatory, antitumour, gastroprotective, hepatoprotective and hypoglycaemic activities, which has great potential in product development. At present, polysaccharides from AM are mainly used as an immune adjuvant, natural plant food supplement to animals, vaccine adjuvant, etc., and have great development prospects.

Generally, polysaccharides from AM have been extensively studied worldwide but the research is very basic and there are still many unsolved puzzles. Firstly, the influence of polysaccharides from AM structure on biological activity is in infancy as it is shown that polysaccharides from AM structures vary with the source and extraction method. Secondly, the activity of polysaccharides is closely related to monosaccharide composition, molecular weight and its distribution, glycosidic linkage, branching degree and conformation. However, all these components in AM have not yet been elucidated. Many studies reported various biological activities (antitumour, immunomodulatory, hepatoprotective, hypoglycaemic, gastroprotective, etc.) of polysaccharides from AM, but there are few studies on its molecular mechanisms. In addition, there are few reports on the development of products containing polysaccharide of AM. It is necessary to establish accurate and reliable methods to confirm the chemical properties of polysaccharides from AM. Plant polysaccharides have great potential for development as delivery systems, so it is necessary to explore the use of polysaccharides from AM as drug carriers to maximize its medicinal value. It is also necessary to perform more *in vivo* experiments to confirm its efficacy and establish safety. In conclusion, the research on the structure, quality control, and activity of polysaccharides from AM should be further strengthened to form a scientific basis for producing novel therapeutic agents, functional foods, and adjuvants to prevent or treat different pathological conditions.
